# Editorial: The role of tumor microenvironment in the development, treatment and prognosis of hepatocellular carcinoma

**DOI:** 10.3389/fphar.2023.1343175

**Published:** 2024-01-04

**Authors:** Haiyu Wang, Hui Liu, Xuming Tang, Jiang Chen, Zhigang Ren

**Affiliations:** ^1^ School of Medicine, Sias University, Xinzheng, Zhengzhou, China; ^2^ Department of Infectious Diseases, The First Affiliated Hospital of Zhengzhou University, Zhengzhou, China; ^3^ The University of Hong Kong, Pokfulam, Hong Kong SAR, China; ^4^ Department of Surgery, Weill Cornell Medicine, Cornell University, New York, NY, United States; ^5^ Department of General Surgery, Sir Run Run Shaw Hospital, Zhejiang University, Hangzhou, China

**Keywords:** tumor microenvironment, hepatocellular carcinoma, development, treatment, prognosis

Primary liver cancer (PLC), including hepatocellular carcinoma (HCC) and intrahepatic cholangiocarcinoma (ICC), and other rare tumors, is the third leading cause of cancer-related mortality (Liu et al., 2021). Of all primary liver cancer, HCC is the most common type. At present, liver resection and transplantation are still the most effective treatments for early-stage liver cancer, but they are not suitable for most liver cancer patients who have been diagnosed at an advanced stage (Yang et al., 2020). While targeted therapy and immunotherapy have made major advances in therapy, HCC still has a particularly poor prognosis (Duchemann et al., 2022). Therefore, a better understanding of the molecular mechanisms underlying HCC progression and effective therapeutic strategies against it are of critical importance (Li et al., 2020).

Evidence has accumulated that the tumor microenvironment (TME) plays a key role in fostering or restraining tumor development (Blanco-Fernandez et al., 2021). The TME, which includes molecular, cellular, extracellular matrix, and vascular signaling pathways, is a complex ecosystem (Huang et al., 2023). The interaction between TME and cancer cells can enhance the malignant properties of tumors, including proliferation, metastasis, and therapy resistance (Hosaka et al., 2020; Tian et al., 2021). In addition, TME can also lead to abnormal angiogenesis and promote immunosuppression (Chen et al., 2023). Therefore, further study of TME of HCC may be helpful for the development of therapeutic methods for HCC.

In this Research Topic, we include 24 original research papers that focus on HCC genes microenvironment, immune microenvironment, metabolic microenvironment, and gut microbial microenvironment, 4 review articles that summarize and critically assess the latest exploration of the role of TME in the development, treatment and prognosis of HCC, and 1 case report that clarify the pathogenesis of intrahepatic mucinous cholangiocarcinoma. We hope that these evidences will further our understanding of the pathogenesis of HCC and provide novel strategies for its treatment.

Macropinocytosis, a type of endocytosis that involves the non-specific uptake of dissolved molecules, has been shown to contribute to HCC pathology. However, its biological mechanism remains unknown. Ding et al. identified 27 macropinocytosis-related genes from 71 candidate genes using bioinformatics and constructed the prognostic signature using seven MPC-related differentially expressed genes (GSK3B, AXIN1, RAC1, KEAP1, EHD1, GRB2, and SNX5) through LASSO Cox regression. This risk model could effectively predict HCC patient prognoses and suggest changes in the immune microenvironment during the disease process.

Anoikis, a novel programmed cell death, has received considerable attention because of its involvement in the progression of human malignancies. Zhang et al. found that anoikis-related genes (ANRGs) were dysregulated in HCC, with a low frequency of somatic mutations and associated with prognosis of HCC patients. Then, they selected nine ANRGs (NQO1, ETV4, BSG, HMGA1, DAP3, PBK, BIRC5, PLK1, and EZH2) to construct a risk score signature based on the LASSO model. The signature presented a strong ability of risk stratification and prediction for overall survival in HCC patients. Similarly, Chen et al. also selected out 3 genes (EZH2, KIF18A and NQO1) and constructed a prognostic model, achieving a good predict performance. Moreover, the results showed that the high-risk group have higher sensitivity to 5-fluorouracil, doxorubicin and gemcitabine.

Copper is an essential trace element that acts as a cofactor in various enzyme active sites in the human body. Intracellular copper homeostasis requires a complex system. It has shown considerable prospects for intervening in TME by regulating copper homeostasis and provoking cuproptosis. Ma et al. constructed a five-gene scoring system in relation to copper homeostasis and cuproptosis through applying LASSO and multivariate Cox regression in TCGA and ICGC datasets, which could forecast clinical prognosis, the TME changes and assist in choosing therapy strategies for HCC patients. Cuproptosis, as a novel cell death pathway dependent on cellular copper ions and ferredoxin 1 (FDX1). Quan et al. founded high FDX1 expression significantly enhanced survival of patients with HCC from the TCGA dataset, and natural killer cells, macrophages, and B cells were significantly enhanced, and PD-1 expression was low in the high-FDX1 tumor tissues. Meanwhile, a high expression of FDX1 decreased cell viability, proliferation and migration of tumor cells. And HepG2 cells with FDX1 expression are sensitive to Cu2+. This study reveals that cuproptosis and tumor immune microenvironment were together involved in improvement of survival in patients with HCC with a high expression of FDX1. Zhou et al. reviewed the biological processes of copper uptake, transport, and storage in human cells, and highlighted the mechanisms by which copper metabolism affects hepatocellular carcinogenesis and metastasis, including autophagy, apoptosis, vascular invasion, cuproptosis, and ferroptosis. Additionally, they summarized the current clinical applications of copper metabolism-related drugs in antitumor therapy. Bao et al. was designed to determine the effect of glutamine metabolism on HCC heterogeneity. They classified HCC into three molecular subtypes according to glutamine family amino acid metabolism process genes. Among them, C1 showed the worst survival rate and the highest immune score and immune cell infiltration. A six-gene model for prognostic and immunotherapy responses was constructed among subtypes, which could serve as a predictor of treatment and prognosis in HCC patients.

Liu et al. stratified HCC into three subtypes based on the protein arginine methyltransferases (PRMT)-related genes. 11 PRMT-related genes were enrolled to establish prognostic model, which presented with high accuracy in predicting the prognosis of two risk groups in the training, validation, and immunotherapy cohort, respectively. Further, the sensitivity of 72 anticancer drugs was identified using prognostic risk model. Wang et al. explored the role of PTGES3 in 33 types of tumors and depicted the potential immune-related pathways among them. The results demonstrated the prognostic predictive value of PTGES3 in a wide range of cancers, which was also associated with the process of tumor immune infiltration. High PTGES3 expression was related to the infiltration of Th2 subsets of CD4^+^ T cells and immune checkpoint-related genes in most cancers, especially in HCC. Zhang et al. established a novel ubiquitin-proteasome system (UPS)-based prognostic risk model in HCC, including seven UPS-based signatures (ATG10, FBXL7, IPP, MEX3A, SOCS2, TRIM54, and PSMD9). Individuals with HCC with high-risk scores presented a more dismal prognosis, larger tumor size, advanced TNM stage, and tumor grade than those with low-risk scores. In addition, obvious immune cell infiltration and sensitive drug response were identified in low-risk patients.

Tumor purity takes on critical significance to the progression of solid tumors. Zhao et al. identified a total of 26 tumor purity–associated genes with differential expression and ultimately identified ADCK3, HK3, and PPT1 as the prognostic genes for HCC. HCC patients exhibiting higher ADCK3 expression and lower HK3 and PPT1 expressions had a better prognosis, high tumor purity, high immune score, high stromal score, and high ESTIMATE score. The dysregulation of RNA splicing is a major event leading to the occurrence, progression, and drug resistance of cancer. Li et al. identified 75 differentially expressed prognosis-related genes from 215 RNA splicing-related genes, and a prognostic model incorporating thioredoxin like 4A (TXNL4A) was identified using least absolute shrinkage and selection operator regression analysis. Chen et al. identified that TAF1B (TATA Box Binding Protein-Associated Factor) was highly expressed in HCC and associated with poor prognosis in HCC patients. TAF1B depletion modulated nucleolar stress and apoptosis in HCC cells through positive and negative feedback from p53-miR-101. RNA polymerase I transcription repression triggered post-transcriptional activation of miR-101 in a p53-dependent manner. In turn, miR-101 negatively feeds back through direct inhibition of the p53-mediated PARP pathway. Tu et al. revealed a novel molecular subtype and prognostic tool based on Foxo signaling pathway signature. According to Foxo prognosis score (FPS) grouping, low FPS exhibited favorable survival, low TMB and anti-tumor activity. Moreover, patients with low FPS were more sensitive to immunotherapy. These results could potentially provide a direction for accurate and effective assessment of potential personalized treatment options and prognostic management for HCC patients.

Natural killer (NK) cells are a type of innate immune cell that recognize and eliminate tumor cells and infected cells, without prior sensitization or activation, playing an indispensable role in antitumor immunity and regulate tumor development. Li et al. identified marker genes of NK cells through Cox and lasso regression by using single-cell RNA-seq data from GEO database. Ten prognosis genes (KLRB1, CD7, LDB2, FCER1G, PFN1, FYN, ACTG1, PABPC1, CALM1, and RPS8) were screened to develop a prognosis model, which demonstrated excellent predictive performance on the training dataset, but also were successfully validated on two independent external datasets. Moreover, molecular docking revealed favorable binding energies between the hub gene ACTG1 and chemotherapeutic drugs. Analogously, Yang et al. also developed an NK cell marker genes-related prognostic signature (NKPS) in the TCGA cohort for risk stratification and prognosis prediction, containing LPCAT1, IL18RAP, SRSF2, ADGRG3, and ADGRE5. The predictive value of the NKPS in prognosis was well validated in different clinical subgroups and three external datasets. In addition, the low-risk group exhibited significantly improved therapeutic benefits, either from immunotherapy or traditional chemotherapy and target therapy. Wang et al. explored the role of macrophage in cellular communication and its effect on the prognosis and immunotherapy of HCC. Through single-cell RNA-seq data from the GSE149614 dataset, they identified 9 cell types, among which macrophage had the highest communication intensity with the rest of the cell types. Based on specifically highly expressed genes of macrophage, they successfully divided HCC patients into three clusters with distinct prognosis, TME, and therapeutic response. Additionally, a risk system was constructed, which provided a potential reference index for the prognostic target and preclinical individualized treatment of HCC. Immunogenic cell death (ICD) plays an important role in the development of cancers. Xiang et al.attempted to explore the role of ICD in the prognosis of HCC. They established a prognostic model including six ICD associated genes for HCC, which may deepen our understanding of ICD and guide therapy for HCC patients. Yang et al. developed and verified a new signature comprising immune- and lipid metabolism-associated markers. This signature can be applied to survival prediction, individualized chemotherapy, and immunotherapeutic guidance for patients with liver cancer. They also provided potential targeted therapeutics and novel ideas for the immune evasion and progression of HCC.

Despite being the standard therapy for advanced HCC, sorafenib frequently encounters resistance, emphasizing the need to uncover the underlying mechanisms and develop effective treatments. Chen et al. highlighted the crucial interplay between the TME, cancer stem cells (CSCs), and epithelial-mesenchymal transition in the context of sorafenib resistance, and proposed that new therapeutic strategies must consider the effects of TME and CSC activation in order to effectively overcome sorafenib resistance in HCC. Cabozantinib (CNB) is a TKI with multiple targets implicated in tumor pathology, including tumor growth, metastasis, and angiogenesis. However, due to the lack of effective targeted drug delivery, its therapeutic effect is poor. Therefore, there is a need to develop a robust and controlled drug delivery system. Bhattacharya et al. developed a promising drug delivery system, consisting of CNB-loaded nanoparticles made from Poly D, L-lactic-co-glycolic acid, and Polysarcosine (CNB-PLGA-PSar-NPs). They found CNB-PLGA-PSar-NPs were effective in inducing apoptosis in HepG2 cell, and observed *in vivo* antitumor activity was well reported in SCID female mice.

Zhou et al. screened afatinib-associated differential expressed genes based on transcriptomic data of HCC patients and used the Genomics of Drug Sensitivity in Cancer 2 database to select small-molecule drugs targeting genes. They founded that ADH1B is a key afatinib-related gene, which is associated with the immune microenvironment and can be used to predict the prognosis of HCC. It is also a potential target of candidate drugs (panobinostat, oxaliplatin, ixabepilone, and seliciclib). Zou et al. demonstrated that nanosecond pulsed electric fields (nsPEFs) have an excellent ablation effect in HCC mice. Compared with normal mice, the gut microbiome diversity of HCC mice was increased and the serum metabolism was significantly altered in nsPEFs group, suggesting that gut microbiome and serum metabolites may participate in the prognosis of HCC ablation. Exosomes are crucial therapeutic agents for a variety of illnesses, such as cancer and autoimmune diseases. Ghaffari et al. reviewed biogenesis of exosome, types of exosomes, and exosome isolation methods. They also discussed the role of exosomes in the development of cancer, exosome-based tumor suppression strategies, the use of exosomes as carriers in the treatment of hematological malignancies. Finally, this study summarized the challenges and limitations of the therapeutic use of exosomes.

For most patients, radical resection, which is advantageous because of its high surgical resection rate and low mortality rate, is the preferred treatment. Unfortunately, the 5-year recurrence rate after radical resection of liver cancer is still high at 60%–70%, and the overall survival rate is still low. Therefore, the prognostic factors and prognostic models after curative resection of HCC patients are urgently needed. Yang et al. constructed a pre-to postoperative alpha-fetoprotein ratio-based nomogram to predict tumor recurrence, and achieved good prediction function. While, Yang et al. founded corona enhancement and MVI could be used to characterize patients with macrotrabecular-massive (MTM)-HCC and predict their prognosis for early recurrence and overall survival after surgery.

HCC often occurs in the setting of chronic liver disease or cirrhosis, while liver disease such as viral hepatitis, alcohol induced liver disease, and non-alcoholic fatty liver disease is a major risk factor for the development of HCC and has been demonstrated to alter the immune microenvironment. Therefore, Brown et al. summarized the liver immune microenvironment, as well as the relative effect of liver disease on the immune microenvironment. In addition, they reviewed how changes in the immune microenvironment can lead to therapeutic resistance, as well as highlight future strategies aimed at developing the next-generation of therapies for HCC.

ICC is a highly aggressive primary liver cancer, with increasing incidence worldwide. Effective first-line treatments for advanced ICC patients are currently limited. Therefore, Huang et al. aimed to assess the efficacy and safety of programmed death-1 (PD-1)/programmed death-ligand 1 (PD-L1) inhibitors in combination with gemcitabine/cisplatin (GC) and lenvatinib as first-line treatment in advanced ICC patients. They included 51 advanced ICC patients, among whom 25 patients were administered with PD-1/PD-L1 plus lenvatinib and 26 patients were administered with PD-1/PD-L1 plus GC. The results showed PD-1/PD-L1 inhibitors in combination with lenvatinib or GC all demonstrated significant efficacy and safety as first-line treatment in patients with advanced ICC. As for patients who refuse or are intolerant to chemotherapy, PD-1/PD-L1 plus lenvatinib would be recommended. Intrahepatic mucinous cholangiocarcinoma (IMCC) is a rare subtype of ICC. Limited data describe the genetic characteristics of IMCC and insights on its pathogenesis are lacking. Zeng et al. employed somatic mutations, transcriptome, proteome and metabolome of tumor tissue obtained from a case of IMCC to clarify the pathogenesis of IMCC. A total of 54 somatic mutations were detected. The results showed the genes consistently upregulated at the transcription level and in the proteome were enriched for mucin and mucopolysaccharide biosynthesis, for cell cycle functions and for inflammatory signaling pathways. Transcription factor ONECUT3 possibly activates the transcription of mucin and mucopolysaccharide biosynthesis in IMCC.

In conclusion, TME is critical for the development, treatment, and prognosis of HCC. The interaction among HCC genes microenvironment, immune microenvironment, metabolic microenvironment, and gut microbial microenvironment profoundly affects the growth, metastasis, drug resistance, and recurrence of tumors. With the increasing number of HCC patients and the in-depth understanding of HCC, research on the HCC TME continues to become increasingly important. In the long term, more and more in-depth studies are needed to unravel the interaction mechanism and find potential therapeutic targets.
